# Genome Sequence, Assembly and Characterization of Two *Metschnikowia fructicola* Strains Used as Biocontrol Agents of Postharvest Diseases

**DOI:** 10.3389/fmicb.2018.00593

**Published:** 2018-04-03

**Authors:** Edoardo Piombo, Noa Sela, Michael Wisniewski, Maria Hoffmann, Maria L. Gullino, Marc W. Allard, Elena Levin, Davide Spadaro, Samir Droby

**Affiliations:** ^1^Department of Agricultural, Forestry and Food Sciences, University of Torino, Turin, Italy; ^2^Centre of Competence for the Innovation in the Agro-environmental Sector, University of Torino, Turin, Italy; ^3^Department of Plant Pathology and Weed Research, Agricultural Research Organization, Volcani Center, Rishon LeZion, Israel; ^4^United States Department of Agriculture – Agricultural Research Service, Kernersville, WV, United States; ^5^Division of Microbiology, United States Food and Drug Administration, College Park, MD, United States; ^6^Department of Postharvest Science, Agricultural Research Organization, Volcani Center, Rishon LeZion, Israel

**Keywords:** postharvest pathology, biocontrol agent, fungi, genome assembly, genome annotation, plant pathogen interactions

## Abstract

The yeast *Metschnikowia fructicola* was reported as an efficient biological control agent of postharvest diseases of fruits and vegetables, and it is the bases of the commercial formulated product “Shemer.” Several mechanisms of action by which *M. fructicola* inhibits postharvest pathogens were suggested including iron-binding compounds, induction of defense signaling genes, production of fungal cell wall degrading enzymes and relatively high amounts of superoxide anions. We assembled the whole genome sequence of two strains of *M. fructicola* using PacBio and Illumina shotgun sequencing technologies. Using the PacBio, a high-quality draft genome consisting of 93 contigs, with an estimated genome size of approximately 26 Mb, was obtained. Comparative analysis of *M. fructicola* proteins with the other three available closely related genomes revealed a shared core of homologous proteins coded by 5,776 genes. Comparing the genomes of the two *M. fructicola* strains using a SNP calling approach resulted in the identification of 564,302 homologous SNPs with 2,004 predicted high impact mutations. The size of the genome is exceptionally high when compared with those of available closely related organisms, and the high rate of homology among *M. fructicola* genes points toward a recent whole-genome duplication event as the cause of this large genome. Based on the assembled genome, sequences were annotated with a gene description and gene ontology (GO term) and clustered in functional groups. Analysis of CAZymes family genes revealed 1,145 putative genes, and transcriptomic analysis of CAZyme expression levels in *M. fructicola* during its interaction with either grapefruit peel tissue or *Penicillium digitatum* revealed a high level of CAZyme gene expression when the yeast was placed in wounded fruit tissue.

## Introduction

The yeast *Metschnikowia fructicola* (type strain NRRL Y-27328, CBS 8853) was first isolated from grapes and identified as a new species by Kurtzman and Droby (2001). The identification was achieved by comparing its nucleotide sequence in the species-specific ca. 500–600-nucleotide D1/D2 domain of 26S ribosomal DNA (rDNA) with a database of D1/D2 sequences from all the recognized ascomycetous yeasts available at that time (Kurtzman and Robnett, 1998), and subsequent entries in GenBank.

Yeasts have been identified by many workers as potential biological control agents suitable for the prevention of postharvest diseases, especially since they are naturally occurring on fruits and vegetables, and exhibit a number of traits that favor their use as fungal antagonists. These traits include high tolerance to environmental stresses (low and high temperatures, desiccation, wide fluctuations in relative humidity, low oxygen levels, pH fluctuations, UV radiation) encountered during fruit and vegetable production before and after harvest, and their ability to adapt to the micro-environment present in wounded fruit tissues, characterized by high sugar concentration, high osmotic pressure, low pH and conditions that conducive to oxidative stress. These traits are especially beneficial for their use as biocontrol agents, since the majority of postharvest decay pathogens are necrotrophic and infect fruit through wounded tissues (Droby et al., 2016; Wisniewski et al., 2016). Additionally, many yeast species can grow rapidly on inexpensive substrates in fermenters, traits that are conducive to their large-scale commercial production and use (Spadaro and Droby, 2016). Moreover, in contrast to filamentous fungi, the vast majority of naturally occurring yeasts do not produce allergenic spores or mycotoxins, and have simple nutritional requirements that enable them to colonize dry surfaces for long periods of time (Spadaro et al., 2008).

Significant progress has been made in the development, registration and commercialization of postharvest biocontrol products (Droby et al., 2009, 2016) and a variety of different biocontrol agents have reached advanced stages of development and commercialization. “Shemer,” based on the yeast *M. fructicola* (Droby et al., 2009), is one of the commercial products that has reached the market.

Several studies have documented the biocontrol efficacy of *M. fructicola* and its ability to prevent or limit the infection of harvested products by postharvest pathogens (Karabulut et al., 2003, 2004; Spadaro et al., 2013). Similar to other postharvest biocontrol agents, *M. fructicola* exhibits several modes of action to achieve its ability to act as an antagonist. Like its sister species *M. pulcherrima, M. fructicola* produces the red pigment, pulcherrimin, which is formed non-enzymatically from pulcherriminic acid and ferric ions (Sipiczki, 2006). Pulcherrimin has been reported to play a role in the control of *Botrytis cinerea, Alternaria alternata*, and *Penicillium expansum* on apple (Saravanakumar et al., 2008). Enhanced expression of several genes involved in defense signaling, including PRP genes and MAPK cascade genes was demonstrated in grapefruit when surface wounds were treated with *M. fructicola* cells (Hershkovitz et al., 2012). The enhanced gene expression was consistent with an induced resistance response suggesting that induced host resistance plays a role in the biocontrol of *M. fructicola* against postharvest pathogens such as *P. digitatum* (Hershkovitz et al., 2012). *M. fructicola* also exhibits chitinase activity and the chitinase gene, *MfChi*, was demonstrated to be highly induced in yeast cells when cell walls of *Monilinia fructicola*, the causal agent of brown rot in stone fruit, was added to the growth medium. These data suggest that *MfChi* may also play a role in the biocontrol activity exhibited by Metschnikowia species (Banani et al., 2015). Macarisin et al. (2010) demonstrated that yeast antagonists, including *M. fructicola*, used to control postharvest diseases have the ability to produce relatively high amounts of superoxide anions. They also demonstrated that yeast cells applied to surface wounds of fruits produce greater levels of superoxide anions than yeast grown *in vitro* in artificial media.

Several studies have examined differential gene expression during the interaction of the yeast *M. fructicola* with host fruit tissue or with the mycelium of the postharvest pathogen *P. digitatum* (Hershkovitz et al., 2012, 2013). Due to the lack of an assembled genome sequence, de-novo assembly of the transcriptome of *M. fructicola* was performed, which resulted in the identification of 9,674 unigenes, half of which could be annotated based on homology to genes in the NCBI database (Hershkovitz et al., 2013). Approximately, 69% of the unigene sequences identified in *M. fructicola* showed high homology to genes of the yeast *Clavispora lusitaniae.* Thus, the RNA-Seq-based transcriptome analysis generated a large number of newly identified *M. fructicola* yeast genes and significantly increased the number of sequences available for *Metschnikowia* species in the NCBI database. Shotgun sequencing data enabled to construct a draft genome of *M. fructicola* based on Illumina paired-end assembly with ∼7000 contigs that was submitted to Genbank (Hershkovitz et al., 2013).

Details about the structure and annotation of the genomes of yeast biocontrol agents are lacking. Such information would be a valuable tool for analyzing the sequences of putative “biocontrol-related” genes among different species of yeast biocontrol agents, characterizing gene clusters with known and unknown functions, as well as studying global changes in gene transcription rather than just specific, targeted genes. Obtaining full genome sequences would also allow comparative genomic analyses to be conducted among closely related yeast species that do not exhibit antagonist properties (Massart et al., 2015).

In the present study, a whole genome sequence of the 277 type-strain of *M. fructicola* (NRRL Y-27328) was assembled using PacBio technology. Results indicate that the genome of *M. fructicola* (Mf genome) is approximately 26 Mbp and contains 8,629 gene coding sequences. The new assembly resulted in a high quality assembly consisting of 93 contigs – the longest one is 2,548,689 bp – with 439X average genome coverage.

In parallel, the genome of another biocontrol strain of *M. fructicola* (strain AP47) isolated in northern Italy from apple fruit surfaces and used to control brown rot of peaches (Zhang et al., 2010), was assembled by aligning Illumina shotgun sequences (with a genome coverage of 161.8 X), using the genome assembly of the strain 277 as a reference. The mutation rate between the two biocontrol strains of *M. fructicola* was also determined.

## Results and Discussion

### Assembly, Gene Prediction and Functional Annotation of the Genome of *Metschnikowia fructicola* Strain 277

**FIGURE 1:**
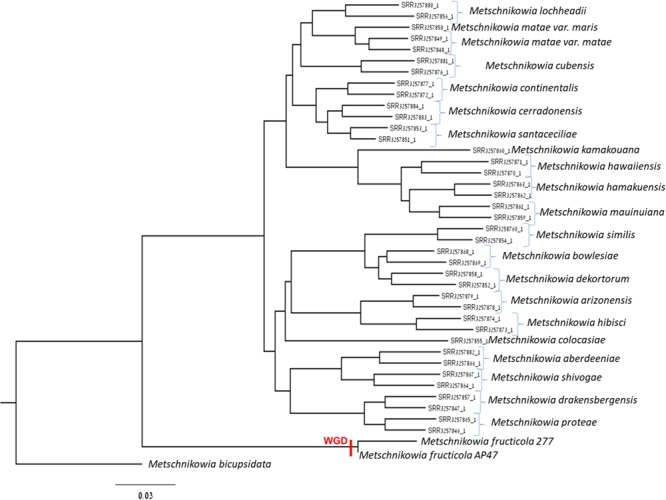
Phylogenetic tree comprised of *Metschnikowia fructicola* 277, *Metschnikowia fructicola* AP47, and other *Metchnikowia* species. The tree was constructed using an assembly and alignment-free method of phylogeny reconstruction (Fan et al., 2015). The whole genome duplication event was indicated on the tree with “WGD.”

A new assembly of the *M. fructicola* (type strain NRRL Y-27328, CBS 8853) genome (Genbank accession ANFW02000000) was constructed using sequence data obtained from the Pacific Biosciences (PacBio) RS II Sequencer. The PacBio genomic sequences were assembled with the HGAP3.0 program (Chin et al., 2013) and yielded a high-quality draft genome consisting of 93 contigs with an N50 of 957,836 bp. The estimated genome size is approximately 26 Mb. Total of 8,629 genes were predicted with MAKER, and 6,262 were successfully annotated with Blast2GO (Conesa et al., 2005) and InterProScan (Finn et al., 2016a,b). The results of assembly, gene prediction and annotation are presented in **Table 1**. In contrast to the previous assembly (Hershkovitz et al., 2013), where 9,674 transcripts were identified, the current high-quality assembly provided a more accurate estimate of the transcript number (8,629) and size of the *M. fructicola* genome. We believe that the current number is more accurate because it was estimated by using the MAKER gene predictor (Cantarel et al., 2008), trained with the transcript sequences obtained by mapping the RNA reads obtained by Hershkovitz et al. (2013) on a high-quality genomic sequence. On the other hand, the 9,674 predicted by Hershkovitz et al. (2013) were obtained by *de novo* assembly with the Trinity software (Grabherr et al., 2011), which can be prone to the overestimation of the number of transcripts (Cerveau and Jackson, 2016). The annotated transcripts are listed in **Supplementary Table S1**, and their sequences, CDSs and protein sequences are presented in **Supplementary Data Sheets S1–S3**. **Supplementary Data Sheet S4** contains the gene coordinates. The main characteristics of the current *M. fructicola* genome assembly and a comparison to the previous assembly (Hershkovitz et al., 2013) are summarized in **Table 1**. Comparative analysis of *M. fructicola* proteins with the other three available closely related genomes of *Clavispora lusitaniae, Candida auris*, and *M. bicupsidata* revealed a shared core of homologous proteins coded by 5,776 genes (**Supplementary Data Sheet S5**). A recently published work describing the phylogeny of strains belonging to Metschnikowia species isolated from the guts of flower-visiting insects (Lachance et al., 2016) allowed us to construct a phylogenetic tree of *Metschnikowia* spp that is based on the fastq raw-data deposited in Genbank (**Figure 1**). The tree was constructed using an assembly and alignment-free method of phylogeny reconstruction (Fan et al., 2015). Interestingly, the phylogenetic analysis showed that the two *M. fructicola* strains described in our study were grouped together and were separate from other *Metschnikowia* species described by Lachance et al. (2016). This difference in phylogeny may be related to evolutionary history and niche colonization of fruit surfaces versus insect guts.

**Table 1:** Summary of the main assembly and annotation features of the genome of the sequenced *Metschnikowia fructicola* strain 277.

	New sequence	Old sequence (Hershkovitz et al., 2013)
Sequencing technology	PacBio	Illumina
Genome size	∼26 Mb	∼23 Mb
Sequencing coverage	20X	700X
Number of contigs	93	8430
Number of large contigs (>100 Kb)	84	2
N50 (base pairs)	957,836 bp	3,784 bp
GC content (%)	45.8%	45.5%
N50 of transcript length (nucleotides)	5033bp	589bp
Number of genes	8,629	15,803
Annotated genes	6,277	–

The GO analysis revealed that 6,262 of the 8,629 identified *M. fructicola* genes were characterized with 4,493 GO terms (**Supplementary Data Sheet S6**). The most common descriptors concerning the cellular component were “Cell” and “Cell Part,” followed by “Organelle,” while “Cellular process” and “Metabolic Process,” followed by “Localization,” “Establishment of Localization,” “Biological Regulation,” “Pigmentation” and “Response to stimulus” were the most common in the biological processes. Regarding the molecular function, the most common descriptors were “Binding” and “Catalytic,” followed by “Transporter.” The same descriptors in the three categories were the most common in the genes characterized in the paper of Hershkovitz et al. (2013).

### Utilization of *M. fructicola* 277 Genome for Reference-Based Assembly of Strain AP47

The assembly of the genome of strain 277 presented here is the most comprehensive and complete assembly for *M. fructicola* to date. This assembly was used as a reference to assemble the genome of the AP47 strain of *M. fructicola*, obtained by Illumina MySeq (161.8 X) shotgun sequencing data (**Table 2**). The reference guided assembly resulted in an N50 of 957,045, which was much higher than the one obtained by *de novo* assembly (**Table 3**). The length of the AP47 genome was similar to the reference strain 277 (∼26 Mb), but had a slightly higher GC content (46.3% compared to 45.8%).

**Table 2:** Sequencing data of the two pair end libraries used to sequence the genome of *Metschnikowia fructicola*, strain AP47.

Sequencing data	Library PE1	Library PE2	Library MP1
Number of raw reads	3717646	2599548	10188012
Number of clean reads	2545140	2546666	9126542
Total length (Mb)	301.257	927.79	2977.528
GC percentage	43% GC	45% GC	43% GC

**Table 3:** *De novo* and reference guided assemblies of the genome of the sequenced *Metschnikowia fructicola*, strain AP47.

	*De novo* assembly^∗^	Reference guided assembly^∗∗^
Sequence length	∼23.3 Mb	∼26.2 Mb
Number of scaffolds	10,173	93
Number of scaffolds > 100 Kb	35	53
Number of scaffolds > 1 Kb	3156	93
N50 (base pairs)	63,477 bp	957,045 bp
G + C content (%)	46.3%	46.3%

*^∗^Obtained with SPAdes (Bankevich et al., 2012). ^∗∗^Obtained with IMR-DENOM (http://mtweb.cs.ucl.ac.uk/mus/www/19genomes/IMR-DENOM/).*

**FIGURE 2:**
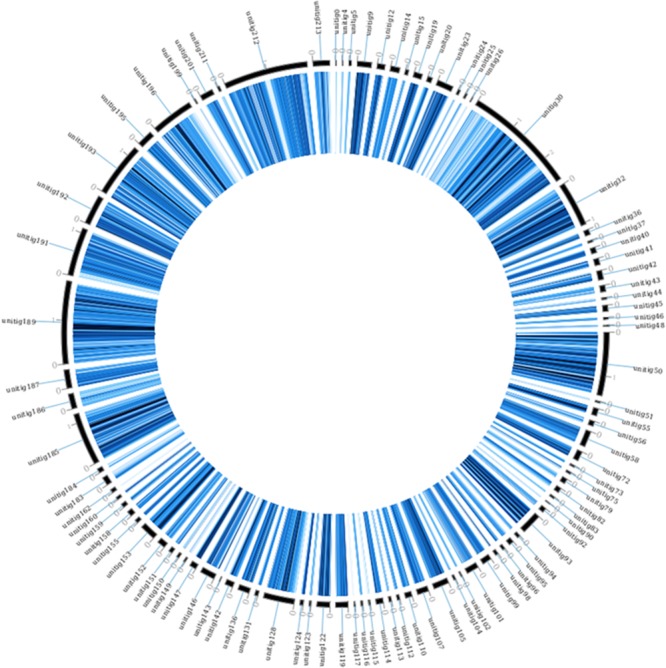
Homologous SNPs of *M. fructicola* strain AP47, mapped on the strain 277 genome. The figure was obtained using circoVCF tool (Drori et al., 2017).

The assembly presented here was also compared to the AP47 strain assembly using a SNP calling approach. Results of this analysis are presented in **Table 4**, and the complete vcf is found in **Supplementary Data Sheet S7**. Considering only homozygous polymorphisms, a total of 546,356 SNPs, 11,987 insertions and 5,959 deletions were identified. Among these mutations, 185,649 were in coding regions, and the vast majority of the variations (135,616) were silent. However, 50,822 were missense mutations, and 212 were nonsense mutations. The differences with strain AP47 were mapped on strain 277 and presented in **Figure 2**.

**Table 4:** Number of mutations in the genome sequence of *M. fructicola* strain AP47, compared to the reference genome of *M. fructicola* strain 277, and their predicted effect and impact on coding sequences.

Number of mutations	564,302
SNPs	546,356
Insertions	11,987
Deletions	5,959
Variant rate	1 variant every 46 bases
**Predicted mutation effect**	
Silent	135,884
Missense	49,794
Nonsense	212
**Mutation impact**	
High	2,023 (0.08%)
Moderate	50,032 (1.97%)
Low	136,810 (5.39%)
Negligible	2,348,195 (92.56%)

The average mutation rate was one every 46 bases, which is exceptionally high in respect to the average reported for other yeast species. For example, the average mutation rate is approximately one SNP every 235 and 269 nucleotides, in *C. albicans* (Hirakawa et al., 2015) and *Saccharomyces cerevisiae*, respectively (Drozdova et al., 2016). The high number of observed mutations may be related to the different geographical origin and host species of the strains. The 277 type-strain of *M. fructicola* (NRRL Y-27328) was isolated in Israel from the surface of grapes, while the AP47 strain was isolated in Italy from the surface of apples.

The strain AP47 Whole Genome Shotgun project has been deposited at DDBJ/ENA/GenBank under the accession MTJM00000000. The version described in this paper is version MTJM01000000.

The D1/D2 region ribosomal region was identified in strain 277 genome by blasting *M. pulcherrima* D1/D2 region on it. Since we observed that none of the identified SNPs were localized in that region, we can confirm with high confidence that both strains 277 and strain AP47 belong to the same species, which is different from *M. pulcherrima* (Kurtzman and Robnett, 1998).

Stress-induced genomic instability has been studied in various yeast and bacteria, under a variety of stress conditions. Stresses were suggested to induce several genetic changes including small changes (one to few nucleotides), deletions and insertions, gross chromosomal rearrangements, copy-number variations and movement of mobile elements (Galhardo et al., 2007).

We suggest that *M. fructicola* as a species could undergo genomic changes in order to survive environmental stresses, in particular on the fruit surface. These changes may have led to evolve mechanisms not only to tolerate stresses, but also to generate large-scale genetic variation as a means of adaptation, giving both *M. fructicola* strains the genetic traits to be successful plant surface colonizers (intact and wounded surfaces) and, possibly, antagonists of fruit pathogens. A second reason of the high polymorphism-rate between *M. fructicola* strains may be the high-mutation rate in the promoters of genes putatively involved in the repair or mutation of the genomic sequences. A list of GO terms related to these processes (**Supplementary Data Sheet S8**) was used to identify 272 annotated genes, and in their promoter sequences the variant rate was of 1/35 bases, against the average of 1/40 in the promoters of the rest of the genomes. The variant rate in the actual transcribed sequence was, however, in line with the rest of the genome (1/66 against 1/67 bases). We also calculated the percentage of these genes showing a putative high impact polymorphism, and 21% of them (57 out of 272) did: this number was slightly higher than the percentage of total genes showing a similar polymorphism (16%, 1,379 out of 8,629).

### Uncommonly Large Genome

The genome of *M. fructicola* was surprisingly large in size, being 26 Mb long. In fact, the most closely related available genomes (*M. bicuspidata, C. auris* and *C. lusitaniae*), are 16 Mb (BioProject PRJNA207846, Riley et al., 2016), 12.5 Mb (BioProjects PRJNA342691 and PRJNA267757, Chatterjee et al., 2015) and 11.9 Mb (BioProject PRJNA12753, Butler et al., 2009), respectively. The most probable explanation for such a genome size seemed to be a whole genome duplication event. To have evidence of this, we searched the genome for homologs, finding 5,132 genes out of 8,629, all in pairs but for 228, which come in groups of three or more copies. This is a high degree of homology, since in the genomes of *M. bicuspidata, C. auris*, and *C. lusitaniae* we found only 71, 69, and 56 homologous genes, respectively.

Ordinarily, after a whole-genome duplication event in yeasts, most of the duplicates of genes situated in low mutation regions are lost, while the ones situated in rapidly evolving regions accumulate mutations and differentiate themselves from their homologs (Fares et al., 2017). We compared the average number of polymorphisms identified between strains 277 and AP47 on homologous and single-copy genes, finding that the first group of genes has a variant rate of 1/65 bases, while for the second group this value is of 1/68. Since divergence between gene copies can also happen at the expression level, so that each copy can be expressed in a different situation and accumulate mutations useful for a specific environmental condition (Fares et al., 2017), the variant rate in the promoters was also checked. Among the promoters of the homologous genes, the average variant rate is of 1/37 bases, while in the single-copy gene promoters it is of 1/45.

Despite the low difference in the mutation rate of single-copy and homologous genes, particularly in the proper gene sequence and not in the promoters, we believe that the available data strengthen the hypothesis of a whole-genome duplication event being responsible for the large genome of *M. fructicola*. This is due principally to the fact that nearly all the homologous genes come in pairs, with only 228 having more than one homolog. The sequencing of other *M. fructicola* strains will undoubtedly be critical to gain further insight on the reasons of this yeast’s large genome.

It should be noted that the strain AP47 has SNPs spread along all the contigs of strain 277 (**Figure 2**). This seems to indicate that the whole genome duplication event occurred in AP47 as well, and that the strains share a common ancestor. This was observed despite the high mutation rate between the strains.

The genomes of the Metschnikowia spp. present in **Table 5** were downloaded from ncbi, to look for others whole-genome duplication events. Since *M. bicuspidata* is the only one of these species to have been fully annotated, it was impossible to look for the whole genome duplication event as has been done with *M. fructicola*. Therefore, we blasted both the transcriptomes of *M. fructicola* and *M. bicuspidata* on all the considered genomes, counting how many of these had matches on different contigs: even if not every transcript had a match, the result of the analysis gave us an idea of the level of homology inside the genomes of interest. In *M. fructicola*, 75% of the transcripts had matches on more than one contig. Furthermore, of the *M. bicuspidata* transcripts with a match on the *M. fructicola* genome, 58% had a match on more than one contig. On the contrary, none of the other analyzed genomes reached a percentage of transcripts mapping on different contigs of 10%. Based on this data, it seems that the whole-genome duplication event is unique to *M. fructicola*. This data correlates well with the high homology level found in the genome, because a high number of homologous genes is commonly associated with relatively recent whole genome duplication events (Lenassi et al., 2013).

**Table 5:** Homology level in different *Metschnikowia* spp. genomes.

	Matched transcripts		Homology level	
	*M. fructicola* transcriptome	*M. bicuspidata* transcriptome	*M. fructicola* transcriptome	*M. bicuspidata* transcriptome
*M. aberdeeniae* (GCA_002370615.1)	39.16%	38.89%	3.64%	4.93%
*M. arizonensis* (GCA_002370875.1)	33.97%	33.3%	4.74%	7.15%
***M. bicuspidata*** (PRJNA207846)	**67.96%**	**100%**	**3.27%**	**9.23%**
*M. bowlesiae* (GCA_002370295.1)	36.77%	38.02%	5.55%	7.26%
*M. cerradonensis* (GCA_002370635.1)	37.66%	38.51%	6.98%	8.1%
*M. colocasiae* (GCA_002370175.1)	39.89%	41.32%	4.71%	6.55%
*M. continentalis* (GCA_002370835.1)	37.46%	38.05%	8.42%	9.37%
*M. cubensis* (GCA_002374405.1)	38.3%	38.98%	6.51%	8.53%
*M. dekortorum* (GCA_002374455.1)	36.46%	38%	5.02%	6.99%
*M. drakensbergensis* (GCA_002370475.1)	39.02%	40.16%	4.1%	5.25%
***M. fructicola***	**100%**	**66.52%**	**74.13%**	**58.23%**
*M. hawaiiensis* (GCA_002370325.1)	40.06%	40.74%	7.52%	9.71%
*M. hibisci* (GCA_002374725.1)	31.71%	29.57%	3.4%	5.91%
*M. kamakouana* (GCA_002374535.1)	38.86%	39.3%	3.67%	5.58%
*M. lochheadii* (GCA_002370915.1)	36.49%	36.3%	7.21%	9.44%
*M. matae* (GCA_002370695.1)	35.07%	35.12%	7.93%	9.56%
*M. mauinuiana* (GCA_002374555.1)	38.63%	39.59%	7.47%	9.04%
*M. proteae* (GCA_002370515.1)	39.65%	40.57%	3.98%	5.83%
*M. santaceciliae* (GCA_002374485.1)	38.08%	38.74%	6.57%	8.4%
*M. shivogae* (GCA_002374645.1)	39.85%	40.19%	3.63%	5.33%
*M. similis* (GCA_002370765.1)	36.93%	38.15%	5.3%	7.5%

*The table is divided in two sections. The left section (Matched transcripts) shows the percentage of *M. fructicola* or *M. bicuspidata* transcripts having a match when blasted on the genome of various *Metschnikowia* spp. The homology level on the right section shows the percentage of matched transcripts which also have a second match on another contig.*

### Carbohydrate Active Enzymes

**FIGURE 3:**
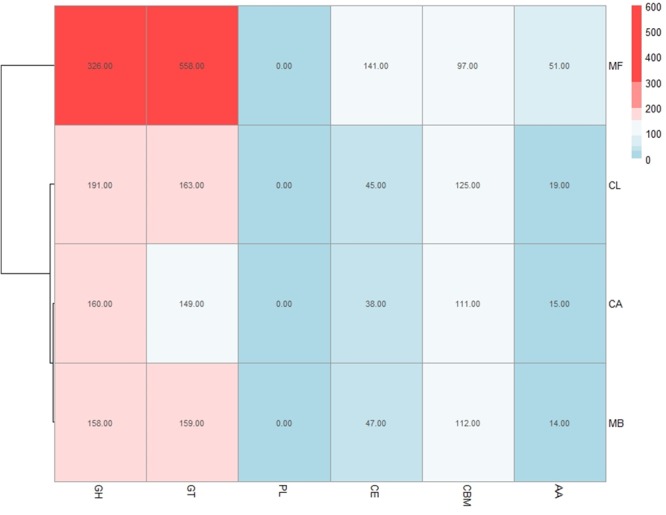
The number of CAZYenzyme genes in each of the 4 sequenced genomes belonging to the Metschnikowiaceae family: Mf – *Metschnikowia fructicola*, Mb – *Metschnikowia bicuspidate*, CL – *Clavispora lusitaniae*, and CA – *Candida auris*. Different classes of CAZYenzyme genes are designated as GH –Glycoside Hydrolases; GT – Glycosyl Transferases; PL – Polysaccharide Lyases; CE – Carbohydrate Esterases; CBM – Carbohydrate-Binding Modules and AA – Auxiliary Activities. The color reflects the relative number of genes in each of the four species as indicated by the scale in the upper right portion of the figure. 28 genes were included in 2 categories, and therefore the sum of the total of **Figure 3** for *M. fructicola* is slightly more than 1,145, which is the reported number of CAZymes.

Plant cell walls consist of a complex network of carbohydrate components, including cellulose, hemicellulose and pectin, as well as a variety of proteins and glycoproteins. These polysaccharides, and other analogous microbial related structural compounds, are targets of carbohydrate-active enzymes (CAZymes) that cleave them into oligomers and simple monomers, which can then be used as nutrients by microorganisms (Cantarel et al., 2009). Bacteria and fungi that are associated with and interact with plants have evolved carbohydrate enzymes strongly linked to the plant environment that these microbes inhabit (Kolton et al., 2013). *M. fructicola* strain 277 MAKER predicted proteins were analyzed with CAT (Park et al., 2010) showing 1,145 putative CAZymes in *M. fructicola* (**Figure 3**). This represents one of the largest number of potential CAZyme genes that have been reported in Ascomycetes (Amselem et al., 2011). In comparison, the genomes of *Botrytis cinerea* and *Sclerotinia sclerotiorum*, two versatile necrotrophic plant pathogens, contain 367 and 346 putative CAZyme genes, respectively, including 106 and 118 clearly related to cell wall degradation (Apweiler et al., 2001). The impressive repertoire of CAZymes in *M. fructicola* thus may play an important role in its nutritional status and ability to colonize plant surfaces as well as being an effective biocontrol control agent. This role becomes particularly important giving that injured fruit surfaces contain a wide variety of simple and complex carbohydrates that can be consumed by pathogens. Despite different studies characterizing the action of some of these genes (Jijakli and Lepoivre, 1998; Friel et al., 2007), the prospective role of CAZymes in the mechanism of action of microbial antagonists is yet to be fully explored. Among the identified CAZymes in *M. fructicola*, 463 have clear assignments to either glycoside hydrolases (GH) or carbohydrate esterases (CE), all involved in fungal cell wall degradation. Two of the aforementioned genes, unitig185_25 and unitig50_23, have a strong resemblance to MfChi (Genbank accession number: HQ113461.1), a *M. fructicola* chitinase which was shown to inhibit *Monilinia fructicola* and *M. laxa in vitro* and on fruit (Banani et al., 2015). A comparison of the number of CAZymes in each of the four annotated genomes belonging to the Metschnikowiaceae family (Mf – *Metschnikowia fructicola*, Mb – *Metschnikowia bicuspidat*a, CL – *Clavispora lusitaniae*, and CA – *Candida auris*) was conducted (**Figure 3**). Mb is a fresh-water fish pathogen, while CL and CA are both human pathogens. Results indicated that the *M. fructicola* genome contained a significantly greater variation and number of CAZyme genes, including glycoside hydrolase (GH), glycosyl transferases (GT) and carbohydrate-binding modules (CBM) family genes (**Figure 3** and **Supplementary Table S2**). The Mf genome contained several unique CAZymes involved in the metabolism of glucans, arabinose, and rhamnogalacturonan that are exclusively associated with terrestrial plant hemicellulose.

### *M. fructicola* Response to *P. digitatum* and to Grapefruit Peel Tissue

The current assembly and genome annotation of Mf enabled us to examine the identification of genes associated with the interaction of Mf with either *P. digitatum* or grapefruit peel tissue and determine the genes that are specific to each interaction.

The transcriptomic RNAseq libraries of Mf, available from BioProject PRJNA168317 (Hershkovitz et al., 2013), were then analyzed. These libraries were constructed from Mf under four different conditions: (1) Mf growing in NYPD broth (control), (2) Mf in contact with *P. digitatum* (Pd) mycelium for 24 h, (3) Mf in contact with *P. digitatum* (Pd) mycelium for 48 h, and (4) Mf in contact with grapefruit peel for 24 h.

**FIGURE 4:**
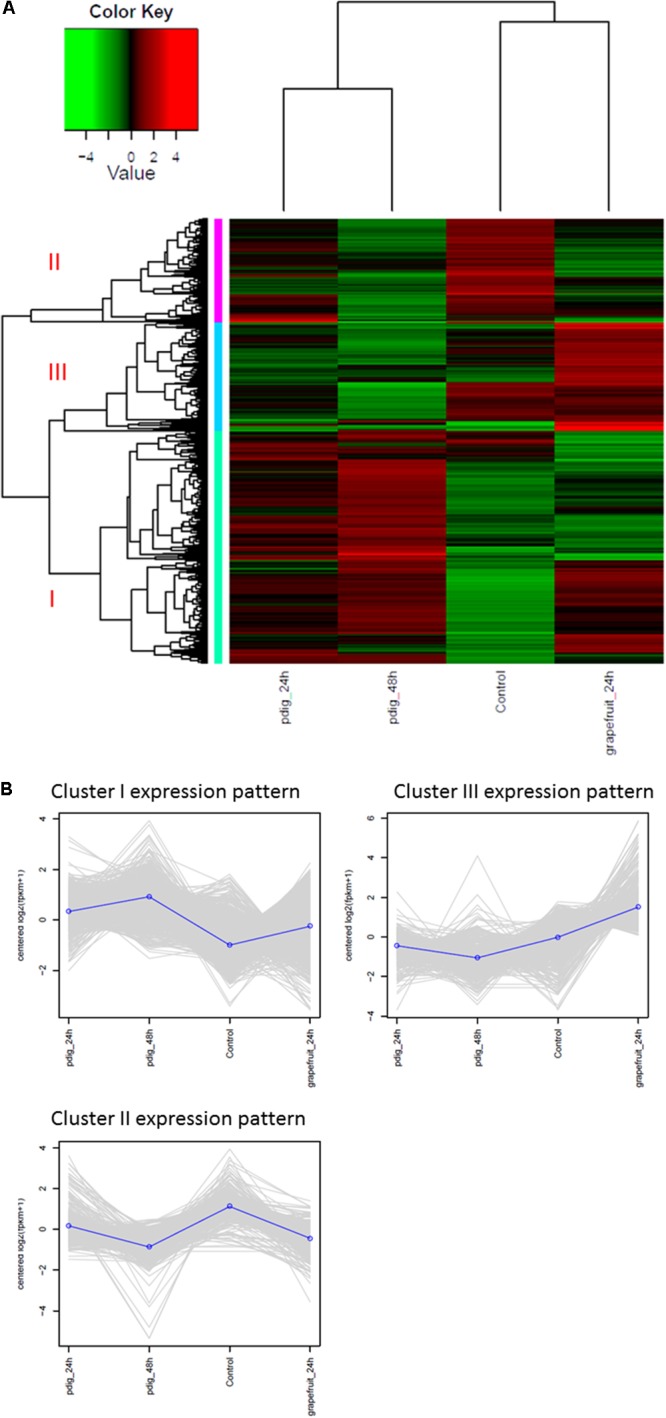
**(A)** Heatmap and expression profile of differentially expressed genes in *Metschnikowia fructicola* (Mf) grown on different substrates. Three clusters were identified. Cluster 1 – genes with higher expression level when Mf was grown in contact with *Penicillium digitatum* (Pd). Cluster2 – genes with higher expression level when Mf was grown in NYPD broth (control). Cluster 3 - genes with higher expression level when in Mf was grown in contact with grapefruit peel. **(B)** The expression profile of the three clusters in response to the different growth conditions.

The analysis of DEGs indicated that gene expression in Mf cells that were in contact with fruit peel tissue or had no contact with fruit tissue (control), was more similar to each other than to gene expression in Mf cells that were in contact with *P. digitatum* mycelia. In total, 2,588 DEGs were identified among Mf cells in contact or not in contact with citrus fruit, peel tissue, and Mf cells that were in contact with *P. digitatum* mycelium (**Supplementary Table S3**). The DEGs could be grouped into three different co-expressed clusters (**Figures 4A,B**).

Cluster1 genes were more highly expressed during contact with *P. digitatum* (Pd) mycelia, relative to cells grown in NYPD broth (control) or on grapefruit peel tissue. We have found 1353 such genes (while only 153 unigenes were found in the previous analysis when using de-novo transcriptome assembly). Cluster 2 genes were more highly expressed in Mf grown in NYPD broth (control) than they were when Mf was in contact with either grapefruit peel tissue or *P. digitatum* mycelium (total of 635 genes). Cluster 3 genes exhibited higher levels of expression when Mf cells were in contact with grapefruit peel tissue, rather than when grown in NYPD broth (control) or in contact with *P. digitatum* mycelium (600 genes).

**FIGURE 5:**
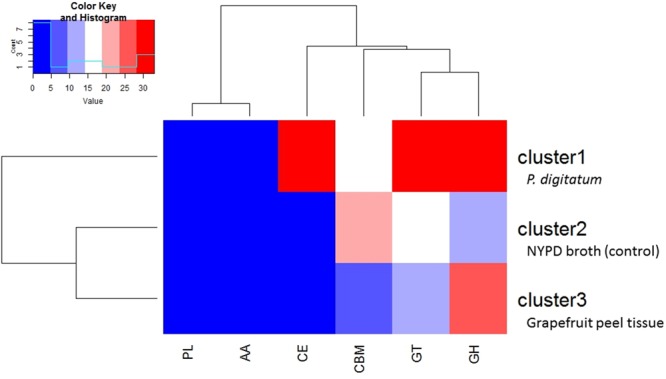
Diagram showing the relative number of genes within each class of CAZymesin each cluster. Cluster1 genes were more highly expressed, relative to cells grown in NYPD broth (control) or on grapefruit peel tissue, when the yeast cells were in contact with *Penicillium digitatum* (Pd) mycelium. Cluster 2 genes were more highly expressed in Mf grown in NYPD broth (control). Cluster 3 genes exhibited higher levels of expression when Mf cells were grown in contact with grapefruit peel tissue. Cluster 3 show also the highest quantity of CAZY enzymes. The color reflects the relative number of genes in each of the clusters as indicated by the scale in the upper left portion of the figure. Different classes of CAZYenzyme genes are designated as GH –Glycoside Hydrolases; GT – Glycosyl Transferases; PL – Polysaccharide Lyases; CE – Carbohydrate Esterases; CBM – Carbohydrate-Binding Modules and AA – Auxiliary Activities.

Transcriptomic analysis of CAZyme expression levels in *M. fructicola* during its interaction with grapefruit peel tissue or *P. digitatum* mycelium when cultured in a PDB medium revealed a high level of CAZyme gene expression when the yeast was placed in wounded fruit tissue (**Figure 5**). These results suggest that CAZyme genes may play an important role in the adaptation of *M. fructicola* to a fruit environment.

### Secondary Metabolite Clusters Present in *M. fructicola*

**FIGURE 6:**
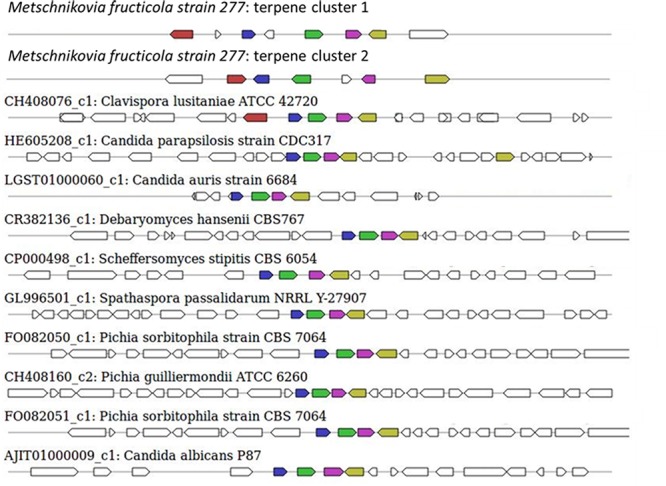
Secondary metabolite clusters producing terpene and their homology with terpene-synthesis clusters in closely related fungi. The terpene synthesis clusters as indicated by antiSMASH3.0 software. The uppermost clusters represent the terpene-synthesis clusters in *M. fructicola* while the terpene-synthesis cluster from other yeasts are shown below.

The sequence of the *M. fructicola* genome revealed that this yeast possesses several secondary metabolite (SM) genes. SMs are known to play an important role in the virulence of many plant pathogens (Namdeo, 2007), but limited knowledge is available about the SM repertoire present in *M. fructicola*. Using antiSMASH (Weber et al., 2015) software, the *M. fructicola* genome was analyzed for the presence of secondary metabolite clusters or homologs of these genes present in related fungi. Twenty-six SM gene clusters were identified in *M. fructicola*, four of which are highly conserved in yeast and other fungi. The remaining 22 clusters could only be designated as putative clusters as similar clusters could not be identified in other fungal genomes using the ClusterFinder algorithm (Cimermancic et al., 2014). These 22 potential clusters included putative saccharide and fatty acid biosynthetic clusters. The analysis of secondary metabolite genes indicated that *M. fructicola* is capable of producing small, potentially bioactive molecules. Two of the identified clusters (**Figure 6** and **Table 6**) code for the production of a terpene that is conserved within *Candida* species. Terpenoid compounds are known to play a significant role in yeast antimicrobial defense mechanism (Hyldgaard et al., 2012). The isoprenoid backbones of these compounds are synthesized by terpene synthases (TSs). The classification of various terpene synthases and their catalytic mechanisms have been recently reviewed (Gao et al., 2012). Although terpenoid SMs have not been previously reported in *M. fructicola*, the genome sequence clearly possesses two gene sequences that encode squalene/phytoene synthases: the transcripts unitig50_211 and unitig147_7.

**Table 6:** Secondary metabolites clusters identified with antiSMASH (Weber et al., 2015) software.

Secondary metabolite cluster type	Transcripts of Mf found in cluster	Location
Terpene cluster	unitig147_4	unitig147
	unitig147_5	15287 – 36642
	unitig147_6	
	unitig147_7	
	unitig147_8	
	unitig147_9	
	unitig147_10	
Terpene cluster	unitig50_207	unitig50
	unitig50_208	578895 – 600250
	unitig50_209	
	unitig50_210	
	unitig50_211	
	unitig50_212	
	unitig50_213	
	unitig50_214	

### YAP Gene Expression in *M. fructicola*

The Yap protein family plays a role in cellular response to oxidative stress (Rodrigues-Pousada et al., 2010) and *M. fructicola* has been demonstrated to have a high tolerance to oxidative stress (Macarisin et al., 2010). An analysis of YAP genes in the *M. fructicola* genome revealed the presence of 14 YAP genes (**Table 7**). In comparison, 7 YAP genes were found in *C. albicans* (BioProjects PRJNA14005 and PRJNA10701), *C. auris* (BioProjects PRJNA342691 and PRJNA267757) and *M. bicuspidata* (BioProject PRJNA207846), while *C. lusitaniae* (BioProject PRJNA12753) had 6. YAP genes are important for resistant to oxidative stress (Macarisin et al., 2010). a feature that could possibly play a role in the ecological fitness and antagonistic activity of *M. fructicola*.

**Table 7:** Yap family genes and homologs identified in the genome of *M. fructicola*.

Systematic name *Saccharomyces cerevisiae*	Homologue in Mf genome	Gene name	Alias(es)	Description
YDR259C	Not found	YAP6	HAL7	Basic leucine zipper (bZIP) transcription factor
YDR423C	Not found	CAD1	YAP2	AP-1-like basic leucine zipper (bZIP) transcriptional activator
YGR241C	unitig192_208	YAP1802		Protein of the AP180 family, involved in clathrin cage assembly
YHL009C	unitig142_42	YAP3		Basic leucine zipper (bZIP) transcription factor
	unitig187_66			
YHR161C	Not found	YAP1801		Protein of the AP180 family, involved in clathrin cage assembly
YIR018W	Not found	YAP5		Basic leucine zipper (bZIP) iron-sensing transcription factor
YJR005W	unitig146_71	APL1	YAP80	Beta-adaptin
	unitig192_37			
YJR058C	unitig122_58	APS2	YAP17	Small subunit of the clathrin-associated adaptor complex AP-2
	unitig50_345			
YLR120C	unitig104_2	YPS1	aspartyl protease,	Aspartic protease
	unitig150_6			
	unitig193_349 unitig32_12			
YLR170C	unitig196_234	APS1	YAP19	Small subunit of the clathrin-associated adaptor complex AP-1
YML007W	Not found	YAP1	PDR4, DNA-binding transcription factor YAP1, SNQ3, PAR1	Basic leucine zipper (bZIP) transcription factor
YOL028C	Not found	YAP7		Putative basic leucine zipper (bZIP) transcription factor
YOR028C	Not found	CIN5	YAP4, HAL6	Basic leucine zipper (bZIP) transcription factor of the yAP-1 family
YPL259C	unitig105_13	APM1	YAP54	Mu1-like medium subunit of the AP-1 complex
	unitig193_251			
YPR199C	Not found	ARR1	ACR1, YAP8	Transcriptional activator of the basic leucine zipper (bZIP) family

### Pulcherrimin Cluster Analysis

Pulcherrimin is a *M. fructicola* metabolite of major interest, since it is involved in the biocontrol action of this yeast (Saravanakumar et al., 2008) and of other biocontrol yeast strains (Castoria et al., 2003). The genes responsible for the biosynthesis of this siderophore were successfully identified only in *B. subtilis* (Randazzo et al., 2016), and an analysis of orthology with proteinortho and blast showed no homology between the *B. subtilis* pulcherrimin gene cluster and the proteins predicted in *M. fructicola*. It is probable that the *B. subtilis* and *M. fructicola* genes involved in pulcherrimin biosynthesis are the product of different evolutionary processes.

## Conclusion

The genomes of two strains of *M. fructicola* (277 and AP47) were sequenced, assembled and compared. The comparison of the two genomes sequences indicated a very high rate of mutation, even though it will be necessary to sequence additional strains to establish if the average mutation rate in *M. fructicola* is intrinsically high, or if the mutation rate identified in the present study is related to the geographical origin and fruit host in which they evolved. The genome size (∼26 Mb) of both *M. fructicola* strains, as well as the rate of mutation, may suggest that *M. fructicola* could undergo genomic changes in order to adapt to plant surfaces, tolerate various environmental stresses and survive under restricted nutritional resources. Its adaptation to plant environment can also be explained by the presence of a relatively large number of secondary metabolites clusters, YAP and CAZymes related genes in the genome.

Another interesting result was the discovery of 1,145 putative CAZymes in the *M. fructicola* genome. These genes could be the target of studies aimed to identify enzymes able to control fungal diseases *in vivo*, to evaluate their potential use as treatments for fruits and plants.

## Materials and Methods

### DNA Extraction

*Metschnikowia fructicola*, Strain 277, (Kurtzman and Droby, 2001) was grown in NYDP (nutrient broth (8 g l^-1^), yeast extract (5 g l^-1^), D-glucose (10 g l^-1^) and chloramphenicol (250 mg l^-1^). One ml of the yeast cell suspension was aseptically transferred from 24 h old starter culture to 250 ml Erlenmeyer flasks and place on an orbital shaker at 160 rpm for 24 h at 26°C. Yeast cells were pelleted by centrifugation at 6,000 rpm, washed twice with sterile distilled water, re-suspended in sterile water to initial volume and the cell suspension concentration was adjusted to 1 × 108 cells ml^-1^.

*Metschnikowia fructicola* strain AP47 was isolated from the carposphere of an apple grown in Piedmont, Northern Italy (Zhang et al., 2010). The strain was stored in tubes of Potato Dextrose Agar and 50 mg/L streptomycin at 4°C. Suspensions of *M. fructicola* AP47 (5 × 10^5^ cells/mL) were inoculated in 500 mL Potato Dextrose Broth (PDB, Difco) and incubated on a rotary shaker (180 rpm) at 24°C for 4 days. Yeast mass was filtered from the culture, frozen in liquid nitrogen and DNA was extracted from 1 g frozen tissue. The final DNA preparation was incubated overnight at room temperature in 490 μl of Tris-EDTA (TE) buffer and 10 μl of DNase-free RNase (10 μg/ml), followed by phenol-chloroform extraction and isopropanol precipitation. Finally, DNA was resuspended in 30 μl TE buffer. DNA concentration and purity were checked by a spectrophotometer (Nanodrop 2000, Thermo Scientific, Wilmington, DE, United States), and the DNA integrity was analyzed by agarose gel electrophoresis (data not shown).

### Sequencing

Strain 277 was sequenced on the Pacific Biosciences (PacBio) *RS* II Sequencer, as previously described (Hoffmann et al., 2013; Pirone-Davies et al., 2015). Specifically, we prepared the library using 10 μg of genomic DNA, that was sheared to a size of 20 kb fragments by g-tubes (Covaris, Inc., Woburn, MA, United States) according to the manufacturer’s instruction. The SMRTbell 20-kb template library was constructed using DNA Template Prep Kit 1.0 with the 20-kb insert library protocol (Pacific Biosciences; Menlo Park, CA, United States). Size selection was performed with BluePippin (Sage Science, Beverly, MA, United States). The library was sequenced using the P6/C4 chemistry on 24 single-molecule real-time (SMRT) cells (8 with BluePippin and 16 without), with a 240-min collection protocol along with stage start.

The genome of *M. fructicola* AP47 was sequenced at the Genomics Platform of the Parco Tecnologico Padano using the Illumina MiSeq technology. Two paired-ends were prepared using Nextera XT DNA Sample Preparation Kit, following the manufacturer’s instructions. Two paired-end (PE) libraries were prepared: PE1 with overlapping paired-end reads and PE2 with non-overlapping paired-end reads. One mate pair library was also prepared, using Nextera Mate Pair Sample Preparation Kit and following the manufacturer’s instructions. Libraries were purified by AMPure XP beads and normalized to ensure equal library representation in the pools. Equal volumes of libraries were diluted in the hybridization buffer, heat denatured and sequenced. Standard phi X control library (Illumina) was spiked into the denatured HCT 116 library. The libraries and phi X mixture were finally loaded into a MiSeq 250 and MiSeq 300-Cycle v2 Reagent Kit (Illumina). Base calling was performed using the Illumina pipeline software. PE1 was composed of 2,1 Gb (330 mean insert size, 43% GC, 35% duplication level). PE2 was composed of 846 Mb (132 mean insert size, 45% GC, 12/duplication level).

All the paired end sequences were trimmed with Trimmomatic v. 0.36 (Bolger et al., 2014) and cleaned with sickle v. 1.33 (Joshi and Fass, 2011) (**Table 2**). The mate pair sequences were trimmed and cleaned with TrimGalore v. 0.4.2^1^.

The genome of *M. fructicola* AP47 was assembled at first with a *de novo* approach, using SPAdes (Bankevich et al., 2012), and then with a reference guided approach using IMR-DENOM^2^, with the strain 277 as the reference.

### Assembly

Analysis of the sequence reads was implemented by using SMRT Analysis 2.3.0. The best *de novo* assembly was established with the PacBio Hierarchical Genome Assembly Process HGAP3.0 program (Chin et al., 2013) using the continuous-long-reads from the four SMRT cells, which contained the longest subreads, with a minimum subread length cutoff of 5000 kb and target coverage of 20X. The resulting HGAP unique contigs (unitigs) were blasted against each other to identify smaller unitigs that show complete overlapping with other larger unitigs. These smaller unitigs were removed from the analysis. Afterward the improved consensus sequence was uploaded in SMRT Analysis 2.3.0. and polished with Quiver using all 24 SMRT cells (Chin et al., 2013).

In total 24 SMRT cells were used, resulting in 93 contigs with 439X average genome coverage. The longest contig comprised 2,548,689 bp.

### Transcriptome Assembly, Gene Prediction and Functional Annotation

RNAseq from previous analysis (Hershkovitz et al., 2013) was used to assemble and predict transcribed regions in the Mf genome. Overall, 6,150 transcripts were identified based on tophat, cufflinks and bowtie2 pipeline as described in (Langmead and Salzberg, 2012).

The transcriptome data, together with the transcripts and proteins sequences available on NCBI for *M. fructicola, M. biscuspidata, C. auris* and *C. lusitaniae*, were used to train the gene predictor SNAP^3^, following the suggested procedure^4^. The augustus gene predictor^5^ was trained with the WebAUGUSTUS web service (Stanke and Morgenstern, 2005), using as data the sequence of the 6,150 transcripts identified with the RNA seq.

SNAP and augustus were then used as a part of the MAKER software (Cantarel et al., 2008) to conduct the gene prediction in the genome. The evidence used were the 6,150 transcripts discovered with the RNA seq and the transcripts and proteins sequences available on NCBI for *M. fructicola, M. biscuspidata, C. auris* and *C. lusitaniae*. The transcripts not coming from *M. fructicola* were included in the MAKER control files as “altest” evidence, which is specifically used for data from species related to the target genome and not from the target itself. The repeat library was constructed following the Basic protocol^6^, and MAKER was launched using the option “correct_est_fusion” in the control files and “-fix-nucleotides” in the command line. MAKER produced a gene coordinates gff3 file, which was used to extract the CDSs from the genome in order to translate them with BioPython (Cock et al., 2009) using the Alternative Yeast Nuclear Code, obtaining the protein sequences. Some of the predicted genes had putative CDSs, which did not start with a start codon and/or did not end with a stop one, and were therefore discarded, with the following exceptions: (i) genes missing the stop codon, localized on the plus filament, which were the last gene of their contig; (ii) genes missing the stop codon, localized on the minus filament, which were the first gene of their contig; (iii) genes missing the start codon, localized on the plus filament, which were the first gene of their contig; (iv) genes missing the start codon, localized on the minus filament, which were the last gene of their contig. The genes of these categories were kept as partial genes.

The proteins were annotated with Blast2GO and Interproscan, using as blast database the fungal fraction of uniprot and swissprot databases (UniProt Consortium, 2017).

The CAT webservice was used to find Pfam modules (Finn et al., 2016b) in the proteins and assign them CAZy families.

Proteinortho v. 5.16 was used to look for homologous proteins in the proteomes of *M. fructicola* 277, *C. auris* (BioProjects PRJNA342691 and PRJNA267757), *M. bicuspidata* (BioProject PRJNA207846) and *C. lusitaniae* (BioProject PRJNA12753).

### Gene Expression Analysis

RNAseq analysis was done using RNAseq data from previous research (Hershkovitz et al., 2013). The RNAseq data number SRA054245 was download from SRA database in NCBI. The RNAseq data was mapped using bowtie (Langmead et al., 2009). Expression quantification was estimated using RSEM software (Li and Dewey, 2011). Differential expression analysis was done using edgeR Bioconductor package (Robinson et al., 2010). Clustering was done using K-mean cluster analysis (Basu et al., 2002) differentialy expressed genes threshold was FDR < 0.05 (Benjamini and Hochberg, 1995) and log fold changes greater than 1 or smaller than -1.

### Phylogenetic Tree

All raw-data sequences of *Metschnikowia* species (Lachance et al., 2016) were downloaded from NCBI using SRAtoolkit (Leinonen et al., 2011) from BioProject ID PRJNA312754. The phylogenetic tree was constructed with an assembly and alignment-free method of phylogeny reconstruction from next-generation sequencing data (Fan et al., 2015).

To place the whole-genome duplication event in the three, we downloaded the genomes of all the considered species, and we used them as databases to blast the full transcriptomes of *M. fructicola* and *M. bicuspidata* (**Table 5**), using blastall v. 2.2.26 with default parameters. We then calculated the percentage of transcripts having a match, and, inside this fraction, the percentage of transcripts having a match on at least 2 contigs.

### Genome Comparison With *M. fructicola* Strain AP47

A SNP calling approach was followed, using bwa mem (Li and Durbin, 2009) to map Illumina reads of the strain AP47 of *M. fructicola* on the assembly of the strain 277. After using samtools view and samtools sort (Li et al., 2009) to obtain a sort.bam file, the following pipeline was used as described by Li (2011) for the SNP calling:

samtools mpileup -guf reference.fa AP47.sort.bam | bcftools view -cg -| vcfutils.pl varFilter -D 200 -Q 20 - > file.vcf

The file AP47.sort.bam was obtained by merging the data from the two Illumina libraries with samtools merge.

The genome of the strain 277 and the gff3 and protein fasta files obtained with MAKER, were used to build a SnpEff (Cingolani et al., 2012) database, and the tool “snpeff eff” was used to evaluate the effect of the homozygous SNPs of the strain AP47. Since *M. fructicola* is a haploid organism, heterozygous SNPs were probably mistakes. The Alternative Yeast Nuclear Code was used to evaluate the effect of missense SNPs on protein sequences.

### Analysis of the Polymorphisms-Related Genes

The variant rate of the genes characterized by gene onthology terms present in **Supplementary Data Sheet S8** was calculated, and the same was done with their promoters. **Supplementary Data Sheet S8** was obtained by selecting all GO terms including the word “repair” or “mutation,” and then removing manually undesired terms (es: “cell wall repair).

The promoter analysis was performed considering as promoter the 1000 bases preceding the genes in the genome, or the 1000 bases following the genes when these were on the antisense strand.

### Analysis of the D1/D2 Region

The primers NL-1 (GCATATCAATAAGCGGAGGAAAAG) and NL-4 (GGTCCGTGTTTCAAGACGG) (O’Donnell, 1993), used by Kurtzman and Robnett (1998) to amplify the D1/D2 region in *S. cerevisiae*, were blasted on the *M. pulcherrima* sequences available on NCBI, so to identify the D1/D2 region. The partial sequence of the large subunit ribosomal RNA gene of *M. pulcherrima* culture-collection CBS:2256 (GenBank: KY108498.1) was therefore downloaded, and blasted on the *M. fructicola* strain 277 genome. We then proceeded to identify the SNPs present in that region in the strains 277 and AP47, looking at both the homozygous and heterozygous SNPs. The blast version used was blastall v. 2.2.26.

### Whole-Genome Duplication Hypothesis

Proteinortho v. 5.16 was used to look for homologous proteins in the proteomes of *M. fructicola* 277, *C. auris* (BioProjects PRJNA342691 and PRJNA267757), *M. bicuspidata* (BioProject PRJNA207846) and *C. lusitaniae* (BioProject PRJNA12753). The variant rate in single-copy and homologous genes was calculated, and the same was done in their promoters.

The promoter analysis was performed considering as promoter the 1000 bases preceding the genes in the genome, or the 1000 bases following the genes when these were on the antisense strand.

### YAP Genes Analysis

The protein sequence of various Yap genes was downloaded from www.yeastgenome.org, and analyzed with Proteinortho v. 5.16 (Lechner et al., 2011), looking for homologs in the proteins predicted for *M. fructicola* strain 277 and in the proteomes of *Candida albicans* (BioProjects PRJNA14005 and PRJNA10701), *C. auris* (BioProjects PRJNA342691 and PRJNA267757), *M. bicuspidata* (BioProject PRJNA207846) and *C. lusitaniae* (BioProject PRJNA12753).

### Secondary Metabolites Cluster Prediction

Secondary metebolites clustering was predicted using antiSMASH website (Weber et al., 2015).

### Pulcherrimin Gene Cluster Analysis

The proteins involved in pulcherrimin biosynthesis in *B. subtilis* (YVNB, YVNA, YVMC, YVMB, YVMA, CYPX; Randazzo et al., 2016) were downloaded from NCBI and used in a proteinortho v. 5.15 analysis with the MAKER predicted proteins of *M. fructicola*, with default parameters. The *B. subtilis* genes of interest were also blasted with blastp (blastall v. 2.2.26) against the predicted proteome of *M. fructicola*, using an *e*-value threshold of 10^-5^.

## Author Contributions

EP and NS performed the bioinformatics analyses and contributed to writing the manuscript. MH and MA performed the PacBio sequencing and contigs assembly. EL contributed in DNA extraction and preparation samples for sequencing. MW, MG, DS, and SD designed the study and wrote the manuscript.

## Conflict of Interest Statement

The authors declare that the research was conducted in the absence of any commercial or financial relationships that could be construed as a potential conflict of interest.

## Footnotes

1 https://www.bioinformatics.babraham.ac.uk/projects/trim_galore/

2 http://mtweb.cs.ucl.ac.uk/mus/www/19genomes/IMR-DENOM/

3 http://korflab.ucdavis.edu/software.html

4 http://weatherby.genetics.utah.edu/MAKER/wiki/index.php/MAKER_Tutorial_for_GMOD_Online_Training_2014

5 http://augustus.gobics.de/

6 http://weatherby.genetics.utah.edu/MAKER/wiki/index.php/Repeat_Library_Construction--Basic

## Supplementary Material

DATA SHEET S1 Fasta file of transcripts of MF.

DATA SHEET S2 Fasta file of CDSs of MF.

DATA SHEET S3 Fasta file of proteins of MF.

DATA SHEET S4 Gff file of MF.

DATA SHEET S5 Proteinortho analysis of *M. fructicola, M. bicuspidata, C. auris* and *C. lusitaniae*.

DATA SHEET S6 Annotation file of MF, produced by Blast2GO.

DATA SHEET S7 Vcf file, obtained by mapping the *M. fructicola* strain AP47 reads on the genome of strain 277.

DATA SHEET S8 List of GO terms related to the mutation or repair of the DNA sequence.

TABLE S1 Annotation of Mf transcripts.

TABLE S2 CAZymes predicted in the *M. fructicola* 277 genome.

TABLE S3 fpkm expression data and statistical differences among conditions analyzed with RNAseq.
